# Enhancing late postmortem interval prediction: a pilot study integrating proteomics and machine learning to distinguish human bone remains over 15 years

**DOI:** 10.1186/s40659-024-00552-8

**Published:** 2024-10-24

**Authors:** Camila Garcés-Parra, Pablo Saldivia, Mauricio Hernández, Elena Uribe, Juan Román, Marcela Torrejón, José L. Gutiérrez, Guillermo Cabrera-Vives, María de los Ángeles García-Robles, William Aguilar, Miguel Soto, Estefanía Tarifeño-Saldivia

**Affiliations:** 1 Gene Expression and Regulation Laboratory (GEaRLab), Department of Biochemistry and Molecular Biology, Faculty of Biological Sciences, University of Concepción, Concepción, Chile; 2 Department of Anthropology and Sociology, Faculty of Social Sciences, University of Concepción, Concepción, Chile; 3 Melisa Institute, Concepción, Chile; 4 Department of Biochemistry and Molecular Biology, Faculty of Biological Sciences, University of Concepción, Concepción, Chile; 5 Department of Computer Science, Universidad de Concepción, Concepción, Chile; 6 BioCell Laboratory, Department of Cellular Biology, Faculty of Biological Sciences, University of Concepción, Concepción, Chile; 7 Department of Anatomy and Forensic Medicine, Faculty of Medicine, University of Chile, Santiago, Chile

**Keywords:** Proteomics, Machine learning, Postmortem interval, Forensic science, Biomarker discovery

## Abstract

**Background:**

Determining the postmortem interval (PMI) accurately remains a significant challenge in forensic sciences, especially for intervals greater than 5 years (late PMI). Traditional methods often fail due to the extensive degradation of soft tissues, necessitating reliance on bone material examinations. The precision in estimating PMIs diminishes with time, particularly for intervals between 1 and 5 years, dropping to about 50% accuracy. This study aims to address this issue by identifying key protein biomarkers through proteomics and machine learning, ultimately enhancing the accuracy of PMI estimation for intervals exceeding 15 years.

**Methods:**

Proteomic analysis was conducted using LC–MS/MS on skeletal remains, specifically focusing on the tibia and ribs. Protein identification was performed using two strategies: a tryptic-specific search and a semitryptic search, the latter being particularly beneficial in cases of natural protein degradation. The Random Forest algorithm was used to model protein abundance data, enabling the prediction of PMI. A thorough screening process, combining importance scores and SHAP values, was employed to identify the most informative proteins for model’s training and accuracy.

**Results:**

A minimal set of three biomarkers—K1C13, PGS1, and CO3A1—was identified, significantly improving the prediction accuracy between PMIs of 15 and 20 years. The model, based on protein abundance data from semitryptic peptides in tibia samples, achieved sustained 100% accuracy across 100 iterations. In contrast, non-supervised methods like PCA and MCA did not yield comparable results. Additionally, the use of semitryptic peptides outperformed tryptic peptides, particularly in tibia proteomes, suggesting their potential reliability in late PMI prediction.

**Conclusions:**

Despite limitations such as sample size and PMI range, this study demonstrates the feasibility of combining proteomics and machine learning for accurate late PMI predictions. Future research should focus on broader PMI ranges and various bone types to further refine and standardize forensic proteomic methodologies for PMI estimation.

**Supplementary Information:**

The online version contains supplementary material available at 10.1186/s40659-024-00552-8.

## Background

The postmortem interval (PMI) is the time from an individual’s death until the discovery of their corpse [1]. Throughout this period, the cadaver undergoes changes influenced by various biotic and abiotic factors, resulting in the decomposition of the cadaver as a dynamic ecological process [2–4]. Precisely determining PMI poses challenges in forensic sciences [5]. PMI estimation accuracy relies on both the time since death and the methodologies used. Research indicates that the precision in estimating intervals up to 24 h is approximately 90% but decreases significantly to around 50% for PMI intervals between 1 and 5 years [6]. For intervals greater than 5 years, referred to hereafter as ‘late PMI,’ there are no standardized methods available [7]. This gap stems largely from the extensive degradation of soft tissues, making traditional methods ineffective [8] and leading to reliance on bone material examinations [9]. Errors in determining PMI can compromise the outcomes of criminal investigations [10], playing a critical legal role in pinpointing the exact timing of a crime. This helps in placing the individuals involved, corroborating witness statements, filtering evidence, and assessing the potential expiration of the statute of limitations for a crime [11]. Therefore, the development of reliable and accurate methods to estimate PMI from skeletal remains is of great importance in forensic science.

In the recent years, proteomics has recently emerged as a promising tool for determining the PMI through the characterization of protein degradation that may serve as biomarkers both in soft tissue and bone [12–16]. This is especially relevant in cases where soft tissues have largely degraded. While bone proteomics at early PMI (< 5 years) has been characterized and conclusive proteomic biomarkers have been suggested, the application of proteomics for late PMI remains underdeveloped.

Franceschetti et al. [17] have recently reviewed the state of late PMI estimation, emphasizing the significant challenges posed by factors like environmental conditions and the lack of standardized molecular methods. While radiocarbon dating (C14) has been the gold standard in archaeology, it is less practical in forensic contexts. Proteomics, however, offers a more feasible approach due to its ability to detect degradation patterns in bone proteins over extended time intervals. Over the past two decades only a handful of studies have aimed to unravel the molecular and biochemical changes that occur in bone during both early and late PMI [18–21].

These studies have shown that: (i) the rate of protein degradation is non-linear, with rapid degradation occurring during the initial months following death; (ii) the type of bone plays a crucial role in predicting PMI, as the morphology and vascularization of bones affect the rate of degradation; and (iii) certain proteins are more resistant to degradation than others. It has been demonstrated that protein degradation accelerates during the first 2 months postmortem, primarily affecting blood-related and cytoplasmic proteins. After this initial phase, the proteome stabilizes, and further degradation slows down [19]. This non-linear pattern of degradation is particularly important in understanding the temporal dynamics of PMI estimation.

Moreover, the stability of the proteome can vary significantly depending on the bone morphology. Bones that are highly porous and vascularized, such as the iliac crest or rib, tend to exhibit faster degradation, whereas bones that are densely cortical and structurally compact, such as the tibia, are more resistant to proteome degradation [20]. The differential proteome stability between bone types underscores the importance of selecting appropriate bone samples for accurate PMI estimation in forensic contexts.

Notably, only one study has differentiated bone proteomes from PMI intervals shorter or greater than 12 years [21]. This work pinpoints that blood-, plasma-related and cytoplasmic proteins are predominant in shorter intervals (< 12 years PMI), while matrix-related proteins dominate at longer intervals (> 12 years PMI). This finding highlights the critical role of bone matrix proteins in PMI estimation, as these proteins interact with hydroxyapatite crystals—the inorganic component of bone. These crystals possess amphoteric properties, enabling them to bind both acidic and basic proteins [22]. As a proof of concept, it has been documented that proteins identified in late PMI are enriched in acidic amino acids [21], suggesting that the interaction between bone matrix and proteins serves as a protective mechanism against degradation. This underscores the potential of matrix-related proteins as reliable biomarkers for late PMI [21]. To develop accurate and reliable strategies for PMI estimation, further comprehensive profiling of matrix-related proteins across different bone types, deposition environments, and late PMI ranges are necessary.

Despite the promising advancements in PMI estimation, there remains a clear need for biomarkers and methods capable of resolving shorter intervals within late PMI. Machine learning (ML) algorithms—which excel in analyzing complex datasets like those generated from proteomics—offer a potential solution for enhancing the accuracy of PMI predictions. In recent years, scientists have successfully applied ML modeling to predict early PMI based on microbial and entomological community succession after death [23]. These studies have achieved prediction accuracies greater than 90%, although they cover PMIs of no longer than 60 days [24–26]. Additionally, research integrating multi-omics (lipidomics, proteomics, and metabolomics) and applying supervised learning mathematical models has managed to accurately identify biomarkers for PMIs of up to 3 years [15]. These studies suggest that combining ML analysis with PMI-suitable biomolecules might be a promising strategy for advancing accurate PMI prediction. However, research on late PMI remains limited, and the identification of reliable biomarkers for this timeframe is still in its early stages.

In this pilot study, we combine LC–MS/MS-based proteomics with machine learning algorithms to differentiate between PMIs of 15 and 20 years. By employing supervised learning on protein abundance data derived from semitryptic peptides, we identified a minimal set of three protein biomarkers that showed high predictive value. Our results suggest that proteomes from tibia samples, which are enriched in bone- and matrix-related proteins, are better suited for accurate late PMI estimation compared to other bone types. These findings highlight the potential of integrating proteomics and machine learning techniques to advance forensic methodologies and improve the accuracy of late PMI prediction.

## Methods

### Acquisition of biological material

Bone remains were extracted from a total of 15 male individuals, aged between 20 and 50 years, whose causes of death, in general, did not involve significant metabolic or infectious diseases. Five of them were obtained from cadaveric donations through the “Body Donors to Science” program of the Department of Anatomy and Legal Medicine at the University of Chile, in accordance with Article 146 of Decree 725 of the Chilean Sanitary Code. The remaining individuals were sourced from exhumations at the General Cemetery of Recoleta (Santiago, Chile), under Article 38 of Supreme Decree 357/70 of the General Regulations of Cemeteries. These legal provisions empower Chilean universities to use cadaveric remains and bone samples from cemeteries for research or educational purposes. Furthermore, the current research underwent scrutiny by the Bioethics and Biosafety Committee of the Faculty of Biological Sciences at the University of Concepción, who approved its execution. Additionally, regarding our experimental design, our work adheres to the principles of the Declaration of Helsinki. From the cadaveric individuals whose time of death was approximately 1 month, a fragment of the left fourth rib was extracted and stored for about 1 year at − 20 °C, categorizing these samples as representing early postmortem interval. On the other hand, individuals from the cemetery represented late postmortem intervals of 15 and 20 years, and fragments of the left rib and tibia were extracted and stored in sealed containers at room temperature. Thus, this study was conducted on a total of 25 bone remains, consisting of 15 rib fragments with PMI < 1, PMI 15, and PMI 20 years, as well as 10 tibia fragments with PMI 15 and PMI 20 years. Since the individuals from the cemetery were retrieved from a burial context, an anthropological assessment of sex and age biological parameters was performed initially, along with the calculation of the Minimum Number of Individuals (MNI) present, to ascertain the characteristics of the sample. The MNI was calculated based on the maximum number of left and right bones present. Sex estimation was carried out using the Phenice method [27] for the coxal bone and the sexual dimorphism parameters for the skull [28]. Age estimation involved the analysis of changes in the pubic symphysis [29], and changes in the auricular surface of the ilium [30].

### Preparation of bone samples and protein extraction

For cadaveric remains, rib fragments were washed with distilled water, and both the adjacent muscular tissue and periosteum were removed using a scalpel. Subsequently, they were dried at 37 °C for one hour. Fragments of 5 × 5 mm were obtained from the cortical region of the body of the rib by a sagittal cut. In the case of skeletal remains, the superficial layer of bone was sanded to eliminate contaminants. 5 × 5 mm rib fragments and 20 × 10 mm midshaft tibia were obtained. The obtained fragments were disinfected with 5% sodium hypochlorite for 15 min, followed by three 30-min washes with nuclease-free water. The fragments were lyophilized for 7 days and pulverized using a mortar. Protein extraction was performed from 100 mg of cortical bone powder, adding 1.5 mL of 99% 2,2,2-trifluoroethanol (TFE) (Sigma Aldrich, 75-89-8) and 1.5 mL of 300 mM Tris(hydroxymethyl)aminomethane (Thermo Scientific, 17926) pH 8.0. The mixture was vortexed and cooled on ice for 10 min. Then, it was sonicated for 5 min with an 80% amplitude in 10-s pulses. Subsequently, it was incubated at 90 °C for 15 min in a dry bath, followed by another 10 min of cooling on ice before centrifuging at 4600×*g* for 10 min at 4 °C. The extracted proteins in the supernatant were quantified using Qubit 4 (Invitrogen), and 30 μg of protein were examined by SDS-Page (5% stacking gel and 10% running gel).

### LC/MS–MS based proteomics

Protein extracts were reduced with 25 mM dithiothreitol (Calbiochem®, 3483-12-3) for 20 min at 37 °C. Subsequently, they were alkylated with 25 mM iodoacetamide (Cytiva, 144-48-9) for 20 min in darkness at room temperature. One volume of 10% 2,2,2-trifluoroethanol (Sigma Aldrich, 75-89-8) was added as a digestion buffer. The samples were digested using a 1:50 w/w ratio of trypsin (Promega, V5111) for 16 h at 37 °C, and the reaction was halted with 0.5% trifluoroacetic acid (Sigma Aldrich, 76-05-1). Peptide cleanup was performed using MCX columns (OASIS, 186000252) following the manufacturer’s instructions.

Finally, vials containing a concentration of 200 ng/µL of peptides in 0.1% formic acid (Thermo Scientific, 85171) were prepared and injected into a nanoUHPLC nanoElute system (Bruker Daltonics), coupled to a timsTOF Pro mass spectrometer (“Trapped Ion Mobility Spectrometry—Quadrupole Time Of Flight Mass Spectrometer,” Bruker Daltonics). The liquid chromatography was carried out using an Aurora UHPLC column (25 cm × 75 μm ID, 1.6 μm C18, IonOpticks, Australia) at 50 °C. Mobile phases A and B were ultrapure water and acetonitrile with 0.1% v/v formic acid, respectively. The percentage of B increased linearly from 2 to 17% in 57 min, to 25% in an additional 21 min, to 35% in 13 more minutes, and then to 85% for a final wash step and re-equilibration. Data collection was performed using TimsControl 2.0 software (Bruker Daltonics) under 10 cycles of PASEF, with a mass range of 100–1700 m/z, a capillary ionization of 1500 V, and a temperature of 180 °C. The TOF frequency of 10 kHz was executed at a resolution of 40,000 FWHM. Protein identification was carried out using the graphical interface of Fragpipe version 18.0 [31] (https://fragpipe.nesvilab.org/) and the MSFragger search engine version 3.5 [32]. The amino acid sequences for Homo sapiens were obtained from Swiss-Prot (accessed September 2022) and used as the database. The established parameters for ion precursor mass tolerance (PMT) and fragment ion mass tolerance (FMT) were 0.05 Da and 20 ppm, respectively. Trypsin enzymatic digestion was assumed to be strict, working on trypsin-like peptides, as well as semi-specific, with semi-tryptic peptides, with a maximum of 2 missed cleavages per peptide and a minimum peptide length of 6 amino acids. Carbamidomethylation of cysteine was considered a fixed post-translational modification, while Oxidation of Methionine (M), N-terminal Acetylation, Deamidation of Asparagine and Glutamine (NQ) were defined as variable post-translational modifications. Philosopher version 4.4 [33], specifically its Peptideprophet tool, was used to calculate the confidence level, considering peptides at a 0.1% false discovery rate. Label-free quantification based on the intensity of MS1 precursors for timsTOF data was performed using Ionquant version 1.8.0 [34], working with default settings. A protein was considered identified when at least one unique peptide was assigned to it.

### Proteome characterization

Descriptive analyses were performed using the NumPy and Pandas libraries in Python. To identify shared and exclusive proteins across different postmortem intervals, we employed UpSetPlot [35], considering proteins identified from both tryptic (tryptic proteins) and semitryptic peptide searches (semitryptic proteins). This analysis allowed us to define a set of ‘representative proteins,’ corresponding to the union of proteins identified by either tryptic proteins or semitryptic proteins. Statistical analyses were performed using *scipy.stats* module from SciPy library [36]. Normal distribution was assessed by Shapiro–Wilk test. Depending on normality, univariate pairwise comparison was performed using t-test or Mann–Whitney U test. Biochemical in-silico profiling was performed using biopython modules *ExPasy*, *SeqIO* and *SeqUtils* [37].

### Machine learning modeling

Both principal component analysis (PCA) and machine learning modeling was performed using several scikit-learn modules [38]. To perform PCA, the protein abundances were first standardized using *StandardScaler*. Subsequently, the PCA analysis was conducted using the *PCA* module from scikit-learn. Cluster search was performed using *KMeans* from *sklearn.cluster* library applied to the principal components. 2D and 3D plots were performed using *Matplotlib* or *Seaborn* libraries [39, 40].

The classification model was constructed using protein abundance based on decision tree and random forest algorithms using the method *RandomForestClassifier* from scikit-learn. The models were refined through a hyperparameters search using the datasets separately for each bone type (tibia or rib) and protein type (tryptic and semitryptic) in late PMI. This search was performed using *RandomizedSearchCV* considering an initial division of the samples in 60% training set and 40% test set. We defined a grid of hyperparameters tailored to the small size of our dataset. The search parameters were set as follows: *n_estimators: randint(100, 500), max_depth:( 5, 10, 15, 20), min_samples_split: randint(2, 3), min_samples_leaf: randint(2, 3), max_features: (‘sqrt’)* and using accuracy metrics for *refit.* In particular, we limited the maximum depth of the trees to 20, as well as the minimum number of samples required for a split and per leaf. Additionally, we used the square root of the total number of features to select features at each node, a commonly recommended parameter for small datasets. This ensures that not all features are considered at each split, increasing the diversity of the trees and enhancing the model’s ability to generalize. The identified hyperparameters were then used to retrain the model. To assess overfitting, we performed 100 iterations of this retraining process, randomly changing the training and test sets in each iteration. Performance metrics, such as accuracy and F1-score, were used for evaluation. For tibia, the best hyperparameters selected were: *n_estimators:*  *158, max_depth:*  *30, min_samples_leaf:* *1, min_samples_split:2, max_features:*  *None*.

For tibia, tryptic and semitryptic proteins, the final hyperparameters found were: *max_depth: 15, max_features: sqrt, min_samples_leaf: 2, min_samples_split: 2, n_estimators: 448*. For rib’s semitryptic proteins, *‘max_depth’: 15, ‘max_features’: ‘sqrt’, ‘min_samples_leaf’: 2, ‘min_samples_split’: 2, ‘n_estimators’: 448*. For rib’s tryptic proteins, *‘max_depth’: 20, ‘max_features’: ‘sqrt’, ‘min_samples_leaf’: 2, ‘min_samples_split’: 2, ‘n_estimators’: 288.*

The identified hyperparameters were then used to retrain the model. To assess overfitting, we performed 100 iterations of this retraining process, randomly changing the training (80%) and test (20%) sets in each iteration. Performance metrics, such as accuracy and F1-score, were used for evaluation. Protein importance for model’s predictive accuracy was calculated by the Mean Decrease in Gini (MDG) and extracted using the attribute *“.feature_importances_”*.

Subsequently, to improve the model’s performance and identify the most important proteins, we conducted a supervised variable screening by combining importance scores and SHapley Additive Explanations (SHAP) values. SHAP values were calculated across the one hundred iterations using the *shap* python library [41] and their distributions were visualized by violin plots. Variable screening was conducted iteratively eliminating proteins that were not informative for the model (importance score) or important for model’s output (SHAP). First, we select all proteins displaying greater than 4% importance score. Then, based on SHAP values distributions, we excluded proteins with median values near zero and negative minimum values. Once a protein set was defined, the model was retrained with 100 iterations, and its performance was evaluated in each iteration.

## Results

### Early and late PMI bone acquisition and protein extraction

Two bone pieces were studied: ribs (n = 5) and tibia (n = 5) at a late PMI of 15 (PMI15) and 20 years (PMI20), as detailed in Table 1. Additionally, we included an early PMI of < 1 year (PMI1) using only rib fragments acquired from male cadaveric donors (n = 5). Proteins were extracted from 100 mg cortical bone powder following an in-house standardized protocol that allowed us to maximize the protein yield for bone proteomics (see “Methods”). Ribs samples showed on average 1.13 ± 0.15 mg (PMI1), 0.63 ± 0.07 mg (PMI15), and 0.82 ± 0.29 mg (PMI20) of total extracted protein. Tibia samples yielded 0.72 ± 0.07 mg (PMI15) and 0.78 ± 0.17 mg (PMI20). The amount of total protein obtained highlights the difficulty of protein extraction from bone tissue and pinpoints that late PMIs yield less amounts of extracted proteins.

**Table 1:** Detailed list and description of bone samples collected for the study

Sample	Individual	Bone	Context	Age	PMI	Storage	Cause of death
MD4C_1	1C	Rib	Cadaveric	31	< 1 year	Frozen	Cardiac stab wound
MD4C_2	2C	Rib	Cadaveric	40	< 1 year	Frozen	Abdomino-pelvic trauma by firearm projectile
MD4C_3	3C	Rib	Cadaveric	21	< 1 year	Frozen	Firearm projectile trauma
MD4C_4	4C	Rib	Cadaveric	22	< 1 year	Frozen	Firearm projectile trauma (thorax)
MD4C_5	5C	Rib	Cadaveric	48	< 1 year	Frozen	Firearm projectile trauma (cervical)
MR1_1C	1R	Rib	Cemetery	35	15 years	Room temperature	Undetermined
MR1_1T		Tibia					
MR1_2C	2R	Rib	Cemetery	35	15 years	Room temperature	Cranioencephalic and facial trauma
MR1_2T		Tibia					
MR1_3C	3R	Rib	Cemetery	43	15 years	Room temperature	Hemorrhagic stroke
MR1_3T		Tibia					
MR1_4C	4R	Rib	Cemetery	43	15 years	Room temperature	Undetermined
MR1_4T		Tibia					
MR1_7C	7R	Rib	Cemetery	49	15 years	Room temperature	Acute subdural hematoma
MR1_7T		Tibia					
MR2_9C	9R	Rib	Cemetery	30	20 years	Room temperature	Polytrauma due to road traffic accidents
MR2_9T		Tibia					
MR2_11C	11R	Rib	Cemetery	39	20 years	Room temperature	Acute posthemorrhagic anemia
MR2_11T		Tibia					
MR2_12C	12R	Rib	Cemetery	42	20 years	Room temperature	Acute pulmonary edema
MR2_12T		Tibia					
MR2_14C	14R	Rib	Cemetery	47	20 years	Room temperature	Asphyxia by hanging
MR2_14T		Tibia					
MR2_15C	15R	Rib	Cemetery	49	20 years	Room temperature	Cardiorespiratory arrest
MR2_15T		Tibia					

### Proteome characterization of ribs and tibia remains

**Fig. 1:**
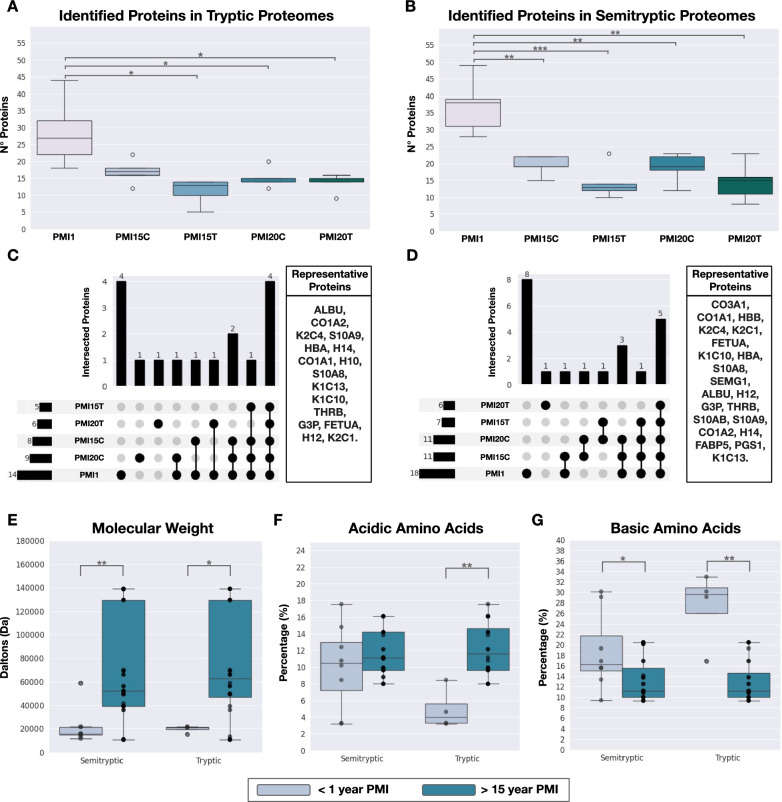
Characterization of proteomes in bone remains. **A** Protein identification using tryptic search. **B** Protein identification using semitryptic search. **C** Comparison of proteomes by PMI and bone segments for proteins identified through tryptic search. **D** Comparison of proteomes by PMI and bone segments for proteins identified through semitryptic search. **E** In silico characterization of molecular weight distribution between PMI < 1 year and PMI > 15 years. **F** Percentage of acidic amino acids in proteins from PMI < 1 year and PMI > 15 years. **G** Percentage of basic amino acids in proteins from PMI < 1 year and PMI > 15 years

Samples were profiled using LC–MS/MS, and the proteome of each sample identified through bioinformatics analysis. The protein identification was carried out using two approaches; (i) a trypsin-specific search, commonly employed in proteomics, generating a proteome referred to as “tryptic proteins or proteome”, and (ii) a nonspecific search, generating a proteome referred to as “semitryptic proteins or proteome”. The semitryptic search was included in our study to account for the expected natural protein degradation in the samples, as the tryptic search could result in the loss of information and bias in abundance estimation. Protein abundances were calculated separately for both tryptic and semitryptic proteins. No significant differences were observed in the total number of proteins identified per PMI between tryptic and semitryptic proteome (Supplementary Table 1). However, early PMIs exhibited a statistically greater number of proteins compared to late PMIs within the same proteome type (Supplementary Tables 2 and 3). As shown in Fig. 1A, for tryptic proteomes, we identified a median of 27 proteins in PMI1, 17 proteins in PMI15 rib (PMI15C), 13 proteins in PMI15 tibia (PMI15T), 15 proteins in PMI20 rib (PMI20C), and 15 proteins in PMI20 tibia (PMI20T). Similar results were observed for identified proteins in semitryptic proteomes (Fig. 1B). To ensure analytical consistency, we established the proteome of each condition as the set of proteins consistently identified across all five biological replicates. Through this approach of consistency filtering, the tryptic proteomes comprised 14 proteins in PMI1, 8 proteins in PMI15 rib (PMI15C), 5 proteins in PMI15 tibia (PMI15T), 9 proteins in PMI20 rib (PMI20C), and 6 proteins in PMI20 tibia (PMI20T) as listed in Supplementary Table 4. Similarly, the semitryptic proteomes included 18 proteins in PMI1, 11 proteins in PMI15C, 7 proteins in PMI15T, 11 proteins in PMI20C, and 6 proteins in PMI20T, detailed in Supplementary Table 5. The proteome analysis among PMIs revealed that most proteins are shared between two or more PMIs (Fig. 1C, D). Specifically, only 4 and 8 proteins were uniquely identified in PMI1 for tryptic and semitryptic proteins, respectively.

To explore whether tryptic and semitryptic proteomes exhibit significant differences, we defined a set of “representative proteins” that included all proteins identified by either tryptic or semitryptic searches across all PMI samples (Fig. 1C, D). A total of 16 representative proteins were identified using tryptic peptides, while 21 were identified using semitryptic peptides. These representative proteins included plasma proteins (ALBU, THRB, FETUA), hemoglobin (HBA, HBB), several collagens and collagen-related proteins (COL1A1, COL1A2, COL3A1, PGS1), keratins (K2C4, K1C10, K1C13), and bone matrix-related proteins (SEMG1, S10AB, S10A9), confirming the bone-specific signature of the obtained proteomes.

Recent hypotheses suggest that proteins persisting in late PMIs (> 12 years) often display higher molecular weights and are enriched in acidic amino acids [21]. To investigate this, we grouped proteomes by early (PMI1) and late PMI stages (PMI15 and PMI20), subtracting proteins present in late PMIs from the early set. Biochemical parameters, such as molecular weight and amino acid composition (acidic and basic), were analyzed (Fig. 1E–G). We observed that proteins in late PMIs, indeed, displayed higher molecular weights (Fig. 1E and Supplementary Table 6) and showed significant enrichment in acidic amino acids (Fig. 1F and Supplementary Table 7). Conversely, the early PMI proteome, identified by both peptide types, was enriched in basic amino acids (Fig. 1G and Supplementary Table 8).

These findings collectively demonstrate that our bone proteomes exhibit distinct identities and biochemical properties dependent on PMI. This led us to question whether these properties could be predictive of PMI in skeletal remains.

### Unveiling global proteomic patterns describing tibia and rib remains

**Fig. 2:**
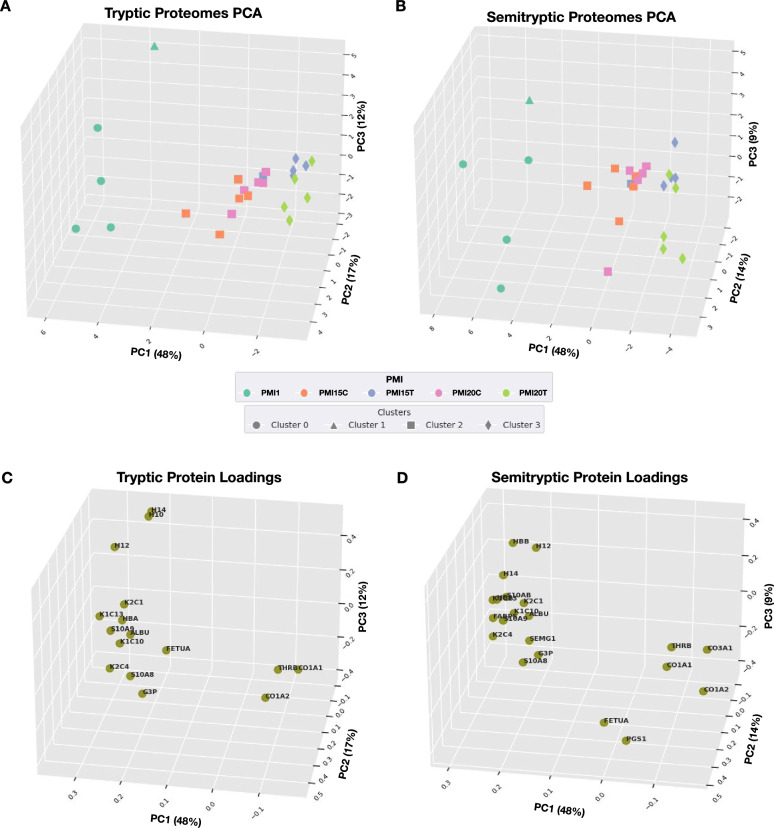
Global visualization of proteomes through principal component analysis. **A** Three-dimensional visualization of the first three principal components for proteins identified through tryptic search. **B** Three-dimensional visualization of the first three principal components for proteins identified through semitryptic search. **C** Variable loadings for the tryptic search. **D** Variable loadings for the semitryptic search

In our quest to elucidate global patterns within proteomic abundance that could potentially inform the PMI, Principal Component Analysis (PCA) was employed as an analytical tool. This statistical method facilitated the effective visualization of major sources of variation within the data, conducted without prior assumptions about sample identity (unsupervised learning). These analyses were coupled with clustering analysis using K-means algorithm for enhancing visualization of sample aggregation through the principal components (PC) as shown in Fig. 2. Our results indicated that protein abundance, calculated based on both tryptic and semitryptic peptides, give rise to similar aggregation and clustering results (Fig. 2A, B). Notably, more than 70% of the variability is explained by the first three principal components (PC1, PC2 and PC3), with PC1 accounting for 48% of the variation, thereby driving the most significant source of variability among samples (Supplementary Table 9 and 10). Surprisingly, we observed that samples do not aggregate by PMI values; instead, it is the type of bone piece (tibia or rib) that drives the clustering (Fig. 2A, B, PC1 in both plots). In both scenarios, PMI1 disaggregates from late PMI ribs, indicating a distinct proteomic pattern. Subsequently, we assessed the variable’s loadings to understand the extent to which each protein influences the observed clustering (Fig. 2C, D). We found that collagen-related proteins (CO1A1, CO1A2, CO3A1, and PGS1) and the plasma protein THRB influence late PMI bone piece aggregation. In contrast, keratins (K2C1, K1C10, K1C13, K2C4), histones (H14, H10, H12), hemoglobins (HBA, HBB), G3P, and bone matrix-related proteins (SEMG1, S10AB, S10A9) are characteristic of early PMI proteomes. Concurrently, we also performed multiple correspondence analysis (MCA) to evaluate whether proteome diversity (presence or absence of proteins) could inform PMI, but this approach did not yield conclusive results (Supplementary Figure 1). These findings suggest that late PMI stages cannot be directly distinguished by PCA, as the bone type has a greater influence on the proteome than the differences in PMI do (15 and 20 years). Additionally, analysis of protein loadings helps to differentiate between early and late PMI stages, although it does not provide insights into specific differences within late PMIs. Based on these insights, we decided to apply ML modeling as an alternative approach to integrate and learn about intrinsic differences between PMI15 and PMI20, which may allow us to distinguish PMI within a 5-year range.

### Machine learning supervised training to accurately predict PMI

We opted for a Random Forest model, an ensemble of decision trees, as our aim was to predict PMI categories. Random Forest is recognized as a robust model for small datasets due to its ability to prevent overfitting by combining multiple decision trees, each built on different subsets of data and variables.

Initially, we focused on modeling late PMI for each bone type—rib and tibia, independently—with the goal of training a model capable of discerning differences within a 5-year PMI range and identifying critical proteins for classification. Once the model was trained and crucial parameters were established (refer to Methods section), we conducted 100 iterations, resampling the training and test sets each time. This process ensured that all samples were included in each set at least once and allowed us to evaluate the model’s consistency across different datasets. These iterations were critical for detecting potential overfitting, as significant variations in performance metrics such as accuracy and F1-score between training and test sets indicated the model’s inability to generalize beyond the training data.

**Fig. 3:**
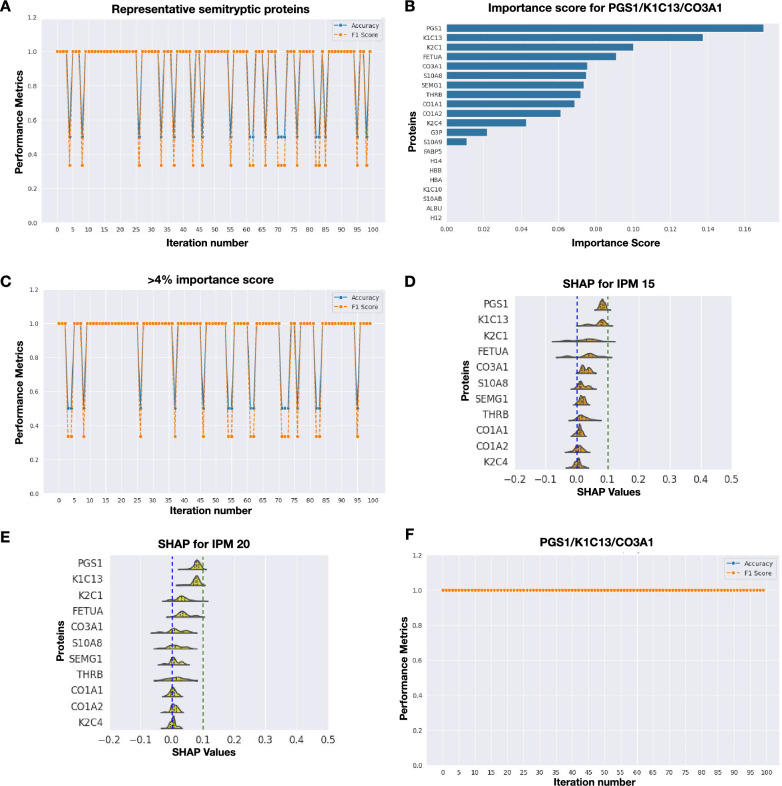
Model optimization via variable screening for tibia late PMI using semitryptic proteins. **A** Initial model performance using the identified hyperparameters and the complete set of representative proteins. **B** Importance score for full set of proteins in model **A**. **C** Model performance after selecting 11 proteins with an importance score greater than 4%. Proteins were then iteratively removed based on their SHAP values. **D** SHAP value distributions for the most important proteins contributing to the classification of PMI 15. **E** SHAP value distributions for the most important proteins contributing to the classification of PMI 20. **F** Final model performance after reducing the set to three key proteins: PGS1, K1C13, and CO3A1

Initial modeling with abundance of full set representative proteins consistently showed better performance for semitryptic proteins in tibia samples (Fig. 3A) compared to tryptic peptides across iterations (Supplementary Figure 2). However, model accuracy oscillated between 100 and 50%, with even lower F1-scores in some iterations (Fig. 3A).

Subsequently, to enhance the model’s performance, we opted to eliminate proteins (referred to as “variables”) that were not informative for the model’s training. Random Forest provides a measure of how informative each protein is through the “importance score”. Additionally, we assessed the contribution of each protein to the model’s output by calculating SHAP values. An iterative selection of the most informative proteins was conducted based on their importance scores and SHAP values. This combined approach enabled the elimination of uninformative or noisy variables, while simultaneously identifying key proteins that contribute to PMI prediction, which aligns with one of the objectives of our study—identifying potential biomarkers for this task.

For the supervised variable screening, the average importance score was calculated from the 100 iterations performed with the full set of representative proteins. As shown in Fig. 3B, for semitryptic proteins from tibia proteomes, the proteins PGS1, K1C13, K2C1, FETUA, CO3A1, S10A8, SEMG1, THRB, CO1A1, CO1A2, and K2C4 displayed importance scores greater than 4%. These eleven proteins were selected for model retraining; however, performance did not improve (Fig. 3C). SHAP distributions for this protein set were plotted for each PMI (Fig. 3D, E). Proteins were then gradually eliminated based on their SHAP values, and model performance was evaluated at each step (Supplementary Figure 3), until optimal and sustained accuracy and F1-scores were achieved with the minimal set of proteins. The supervised variable screening successfully improved the model’s performance, resulting in a consistent accuracy and F1-score of 100% across all iterations (Fig. 3F). The minimal set of proteins sufficient to classify samples from 15- and 20-year PMI was found to be PGS1, K1C13, and CO3A1.

Interestingly, the refinement process using tryptic proteins did not lead to steady improvements in model performance (Supplementary Figure 2). These findings suggest that, in a forensic context, protein abundance estimated from the semitryptic search more accurately reflects bone decomposition than abundance obtained through tryptic search.

Similarly, rib samples modeling consistently demonstrated poor performance for both tryptic proteomes (Supplementary Figure 4) and semitryptic proteomes (Supplementary Figure 5). These findings led to the exclusion of late PMI rib data and tibia tryptic peptides from downstream analysis. At the same time, these results demonstrate that tibia bone and semitryptic protein abundance seems to be reliable predictors for late PMI.

**Fig. 4:**
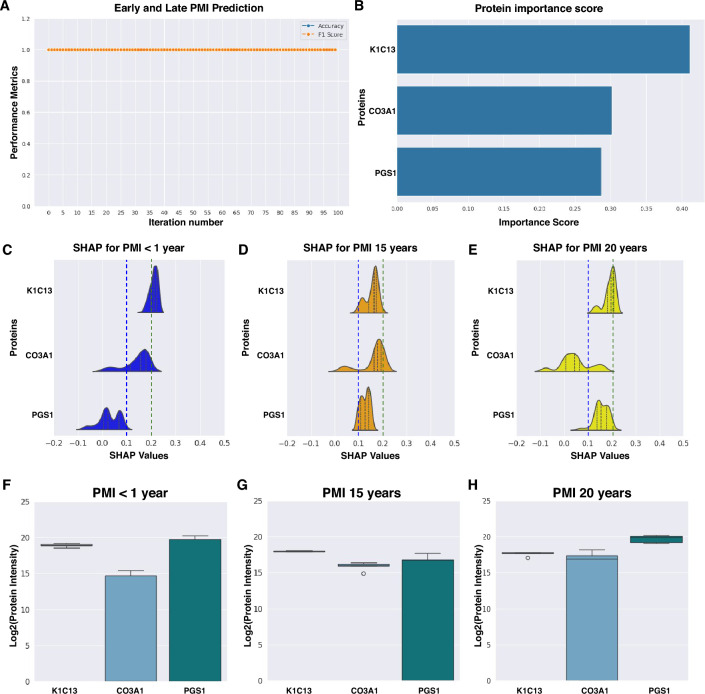
Potential protein biomarkers for distinguishing Early and Late PMI. **A** Prediction performance using proteins K1C13, CO3A1, and PGS1 to classify PMI < 1 year, PMI 15 years, and PMI 20 years. **B** Importance score for the model in **A**. **C**–**E** Distribution of SHAP values for the three protein biomarkers across 100 iterations for rib samples with PMI < 1 year (**C**), tibia samples with PMI 15 years (**D**), and tibia samples with PMI 20 years (**E**). **F**–**H** Semitryptic protein abundance (intensity) for K1C13, CO3A1, and PGS1 across 100 iterations for rib samples with PMI < 1 year (**F**), tibia samples with PMI 15 years (**G**), and tibia samples with PMI 20 years (**H**)

Subsequently, we integrated PMI1 samples into the late PMI tibia modeling to assess the stability performance with an additional PMI category. The model maintained 100% accuracy across iterations, effectively predicting PMI using semitryptic abundances of K1C13, PGS1, and CO3A1 (Fig. 4A, B). Thus, these three proteins constitute potential biomarkers for PMI prediction.

To further explore how the model has learned to differentiate PMIs based on the expression patterns of the critical proteins—K1C13, PGS1, and CO3A1—we analyzed the distribution of SHAP values across the 100 iterations of our final model for these potential biomarkers (Fig. 4C–E). At this point, the SHAP values reflect the contribution of each protein’s abundance to the model’s classification decisions. For instance, K1C13 and CO3A1 show a greater contribution to the classification of PMI1 (Fig. 4C). In contrast, PMI15 classification integrates expression pattern of K1C13, CO3A1, and—to a slightly lesser extent—PGS1 (Fig. 4D). Meanwhile, for PMI20, only K1C13 and PGS1 are critical (Fig. 4E). The semitryptic abundances of these proteins across the different PMIs are provided in Fig. 4F–H, illustrating the specific expression patterns that the model relies on for accurate classification. This analysis clarifies the quantitative shifts in protein abundance required for the model to differentiate between PMI categories, further supporting K1C13, CO3A1, and PGS1 as potential biomarkers for PMI estimation.

## Discussion

This study provides valuable insights into the estimation of late postmortem interval (PMI), addressing three key aspects: (i) the importance of selecting the appropriate bone for proteomic analysis, with the tibia outperforming the ribs; (ii) the advantage of using a semitryptic search to enhance protein identification in degraded samples; and (iii) the value of supervised machine learning (ML) techniques, particularly Random Forest, for predicting shorter intervals within late PMI. These findings underscore the potential of combining proteomics and ML to improve the accuracy of forensic methodologies, while also highlighting the limitations of our study, including the small sample size and experimental design.

Selecting the appropriate bone for proteomic analysis significantly influenced the accuracy of PMI predictions, with tibia outperforming ribs in terms of proteome stability and predictive power. The tibia, a long and highly mineralized bone, exhibited greater resistance to protein degradation compared to the rib, a flat and highly vascularized bone involved in hematopoiesis [42]. This observation aligns with previous findings that emphasize the tibia’s structural compactness and lower vascularization, which protect its proteome from rapid degradation [43]. Studies by Mickleburg et al. also highlight how bone type affects proteome decay, showing that the iliac crest (a flat bone like the rib) is more susceptible to degradation than the tibia in early PMI stages [20]. Our results support this, with rib samples showing greater proteome diversity at PMI1 but a marked decline by PMI15 and PMI20, likely due to their higher exposure to environmental factors such as microbial infiltration [20, 44, 45].

In contrast, the tibia proteomes exhibited lower diversity, particularly at later PMIs, a finding consistent with studies showing that tibia proteins, particularly matrix-related proteins, are more resistant to degradation. This lower diversity does not imply a lack of predictive power; rather, it highlights the importance of focusing on proteins that are more stable over time, particularly those bound to the bone matrix. Our filtering criteria, which focused on proteins consistently identified across all replicates by condition, may have further reduced the diversity of tibia proteomes previously reported [18–20], but ensured consistency and reliability in our analysis. This is crucial for developing reproducible forensic tools, as protein degradation is non-linear and varies depending on bone type and external conditions [21].

Other critical methodological decision in this study was the use of a semitryptic search [46] to enhance protein identification in degraded samples. While most studies rely on tryptic-specific searches for proteomics [18–20], our use of semitryptic searches allowed for the identification of a larger set of proteins and estimate more accurate abundance, particularly in samples where protein degradation is expected, such as those from late PMI. Semitryptic peptides, which only partially conform to trypsin cleavage patterns, increase the sensitivity of protein discovery by capturing degradation fragments that would be missed in tryptic-only searches. This broader search strategy not only enhanced protein detection but also improved predictive accuracy in our models. In tibia samples, the use of semitryptic peptides yielded the most accurate PMI predictions, underscoring their value in forensic proteomics.

On the other hand, the integration of supervised ML modeling, particularly Random Forest, proved essential for differentiating between late PMI intervals. Random Forest models, which aggregate decision trees based on different subsets of data and variables [47], are well-suited to handle the non-linear relationships between protein abundance and PMI. In our study, the use of Random Forest allowed us to identify a minimal set of three key proteins (K1C13, PGS1, and CO3A1) that consistently distinguished between PMIs of 15 and 20 years with 100% accuracy across 100 iterations in tibia samples. Unlike unsupervised methods previously use for PMI prediction such as Principal Component Analysis (PCA) [18–21], which is limited to data visualization and dimensionality reduction [48], Random Forest provides robust predictive power, making it ideal for the complex, multi-dimensional data encountered in bone proteomics.

Our findings align with previous studies applying ML models to early PMI estimation, such as the work by Prieto-Bonete et al. [21], but extend these methods to late PMI. Prieto-Bonete et al. identified proteins that distinguish between PMIs shorter and greater than 12 years, focusing on bone structural proteins and excluding blood-related proteins. In contrast, our approach included a wider range of proteins, including those related to blood and inflammation (e.g., S10A8, S10A9), which may play important roles in bone turnover and postmortem protein stability [49, 50]. The inclusion of these proteins, alongside collagens and other matrix-related proteins (e.g., CO3A1, K1C13, CO1A2, FETUA), proved critical for improving the accuracy of our model. Additionally, the biochemical profiles of proteins in our study, characterized by higher molecular weights and enrichment in acidic amino acids, corroborate previous findings that suggest these characteristics are associated with late PMI [21, 22].

Finally, while our study demonstrates the potential of combining proteomics with ML for late PMI prediction, several limitations must be acknowledged. The small sample size, particularly with regard to tibia and rib samples, restricts the generalizability of our findings. Additionally, our study focused on a narrow PMI range (15–20 years), limiting the scope of the conclusions that can be drawn. Future research should aim to expand the sample size and investigate a broader range of PMIs, as well as include other bone types to further refine and validate the identified biomarkers. Despite these limitations, this study serves as a proof of concept, highlighting the feasibility of integrating proteomics and ML for forensic applications. Our findings lay the groundwork for future research aimed at developing standardized methodologies for late PMI estimation in forensic science.

## Conclusions

Despite its limitations in sample size and the specific PMI range studied, this research provides a significant contribution to the field of predictive forensics by implementing novel experimental approaches that have not been widely explored. Within our experimental framework, we conclude that the use of semitryptic peptides for protein identification in the midshaft tibia, combined with machine learning techniques, significantly improves PMI prediction accuracy. However, to advance toward a generalized methodology for PMI estimation, future research should focus on more comprehensive profiling. This should include a broader range of late PMIs, various bone segments, and an exploration of different deposition contexts. Such efforts will not only deepen our understanding of PMI estimation but also move us toward the standardization of forensic proteomics practices.

## Supplementary Information

Supplementary Figure 1. Global visualization analysis of proteome diversity through multiple correspondence analysis. Accumulated inertias explained by different dimensions and three-dimensional visualization of MCA results for tryptic search and semi-tryptic search.

Supplementary Figure 2. Modeling and variable screening for tibia tryptic proteins selected iteratively based on their importance scores and SHAP values. A. Model using identified hyperparameters and the full set of representative proteins. B. Model using eight proteins displaying > 4% importance score. C. SHAP values for the 4% most important proteins in the classification of PMI 15 years. D. SHAP values for the 4% most important proteins in the classification of PMI 20 years. E. Model using THRB, K2C1, K1C13, FETUA, S10A8, and CO1A2. F. Proteins CO1A2 and FETUA were discarded from the model E. G. Final model using a minimal set of three proteins: THRB, K2C1, and K1C13. H. Importance score for proteins used in model G.

Supplementary Figure 3. Variable screening for tibia semitryptic proteins selected iteratively based on their importance scores and SHAP values. A. Model using identified hyperparameters and the full set of representative proteins. B. Model using 11 proteins displaying > 4% importance score. C. Model using PGS1, K1C13, FETUA, K2C1, CO3A1, THRB, and SEMG1. D. Proteins THRB and SEMG1 were discarded for the model in C. E. Subsequent modeling with the elimination of K2C1. F. Final model using the minimal set of three proteins: PGS1, K1C13, and CO3A1.

Supplementary Figure 4. Variable screening for tryptic proteins in ribs selected iteratively based on their importance scores and SHAP values. A. Model using identified hyperparameters and the full set of representative proteins. B. Model using ten proteins displaying > 4% importance score. C. SHAP values for the 4% most important proteins in the classification of PMI 15. D. SHAP values for the 4% most important proteins in the classification of PMI 20. E. Model using K2C1, K2C4, K1C10, S10A9, K1C13, S10A8, and CO1A2. F. Final model using a minimal set of three proteins: K2C1, K2C4, and K1C10.

Supplementary Figure 5. Variable screening for semitryptic proteins in ribs selected iteratively based on their importance scores and SHAP values. A. Model using identified hyperparameters and the full set of representative proteins. B. Model using eleven proteins displaying > 4% importance score. C. SHAP values for the 4% most important proteins in the classification of PMI 15. D. SHAP values for the 4% most important proteins in the classification of PMI 20. E. Model using K1C10, K2C4, K1C13, K2C1, and CO3A1. F. Final model using a minimal set of three proteins: K1C10, K2C4, and CO3A1.

Supplementary Material 6. Supplementary Tables

Supplementary Material 7. Raw intensities

## Data Availability

The intensity raw files are available as supplementary data.

## Abbreviations

PMI: Postmortem interval
LC–MS/MS: Liquid chromatography–mass spectrometry/mass spectrometry
MNI: Minimum number of individuals
TFE: 2,2,2-Trifluoroethanol
TFA: Trifluoroacetic acid
PCA: Principal component analysis
K-means: K-means clustering algorithm
MCA: Multiple correspondence analysis
ML: Machine learning
SHAP: SHapley Additive exPlanations
PRIDE: Proteomics identifications database
MSFragger: Mass spectrometry Fragger
FDR: False discovery rate
