# Supplementary material for: Enhancing late postmortem interval prediction: a pilot study integrating proteomics and machine learning to distinguish human bone remains over 15 years
Source: Biol Res. 2024 Oct 24;57:75. doi: 10.1186/s40659-024-00552-8 (PMC11515459; doi:10.1186/s40659-024-00552-8)
Supplement: Supplementary file 1 — Supplementary Figure 1. Global visualization analysis of proteome diversity through multiple correspondence analysis. Accumulated inertias explained by different dimensions and three-dimensional visualization of MCA results for tryptic search and semi-tryptic search. [file 40659_2024_552_MOESM1_ESM.pdf]

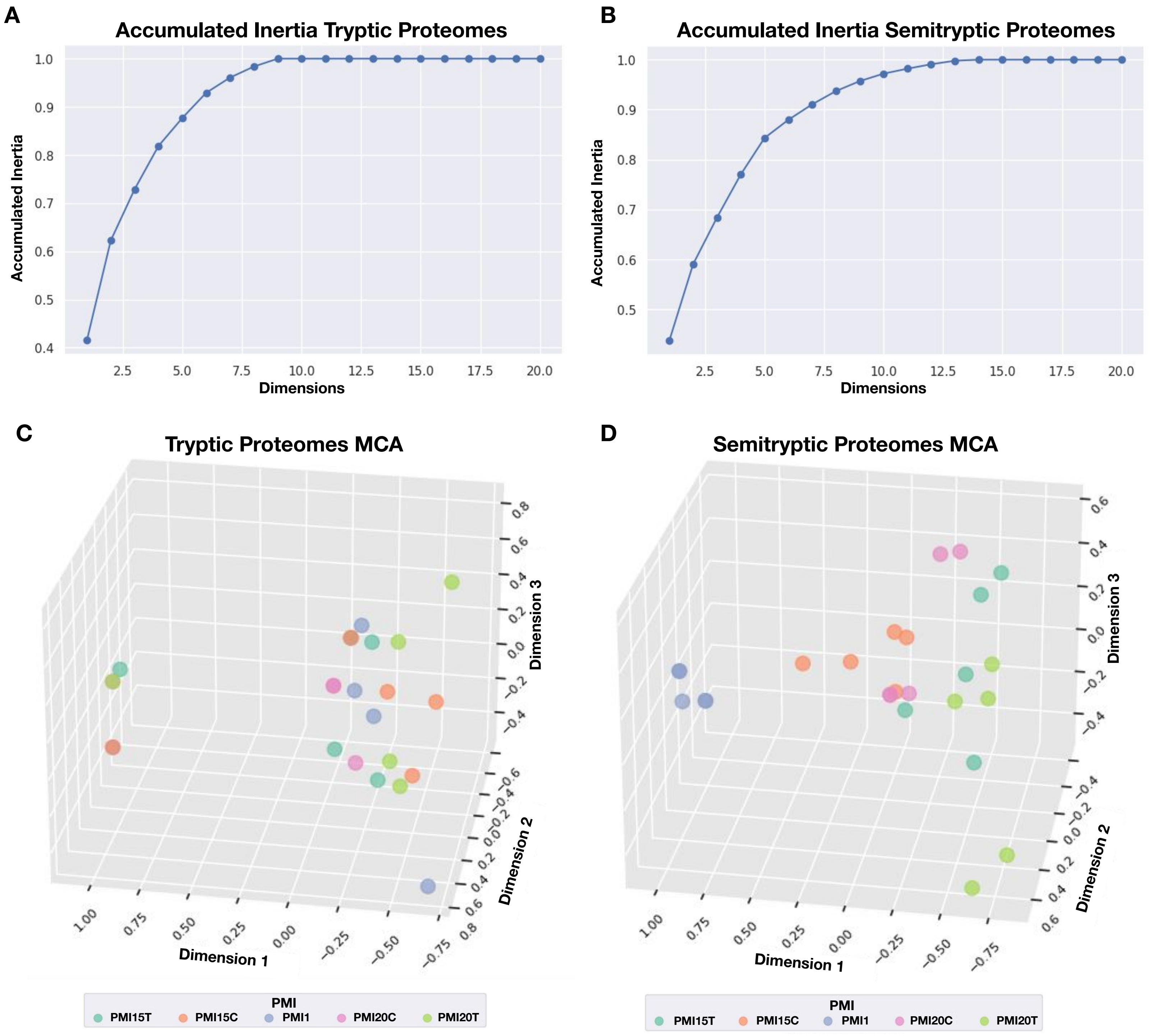

Supplementary Figure 1. Global Visualization Analysis of Proteome Diversity Through Multiple Correspondence Analysis (MCA).

Accumulated inertias explained by different dimensions and three-dimensional visualization of MCA results for tryptic search (A and C) and semi-tryptic search (B and D).
