# Supplementary material for: Enhancing late postmortem interval prediction: a pilot study integrating proteomics and machine learning to distinguish human bone remains over 15 years
Source: Biol Res. 2024 Oct 24;57:75. doi: 10.1186/s40659-024-00552-8 (PMC11515459; doi:10.1186/s40659-024-00552-8)
Supplement: Supplementary file 2 — Supplementary Figure 2. Modeling and variable screening for tibia tryptic proteins selected iteratively based on their importance scores and SHAP values. A. Model using identified hyperparameters and the full set of representative proteins. B. Model using eight proteins displaying > 4% importance score. C. SHAP values for the 4% most important proteins in the classification of PMI 15 years. D. SHAP values for the 4% most important proteins in the classification of PMI 20 years. E. Model using THRB, K2C1, K1C13, FETUA, S10A8, and CO1A2. F. Proteins CO1A2 and FETUA were discarded from the model E. G. Final model using a minimal set of three proteins: THRB, K2C1, and K1C13. H. Importance score for proteins used in model G. [file 40659_2024_552_MOESM2_ESM.pdf]

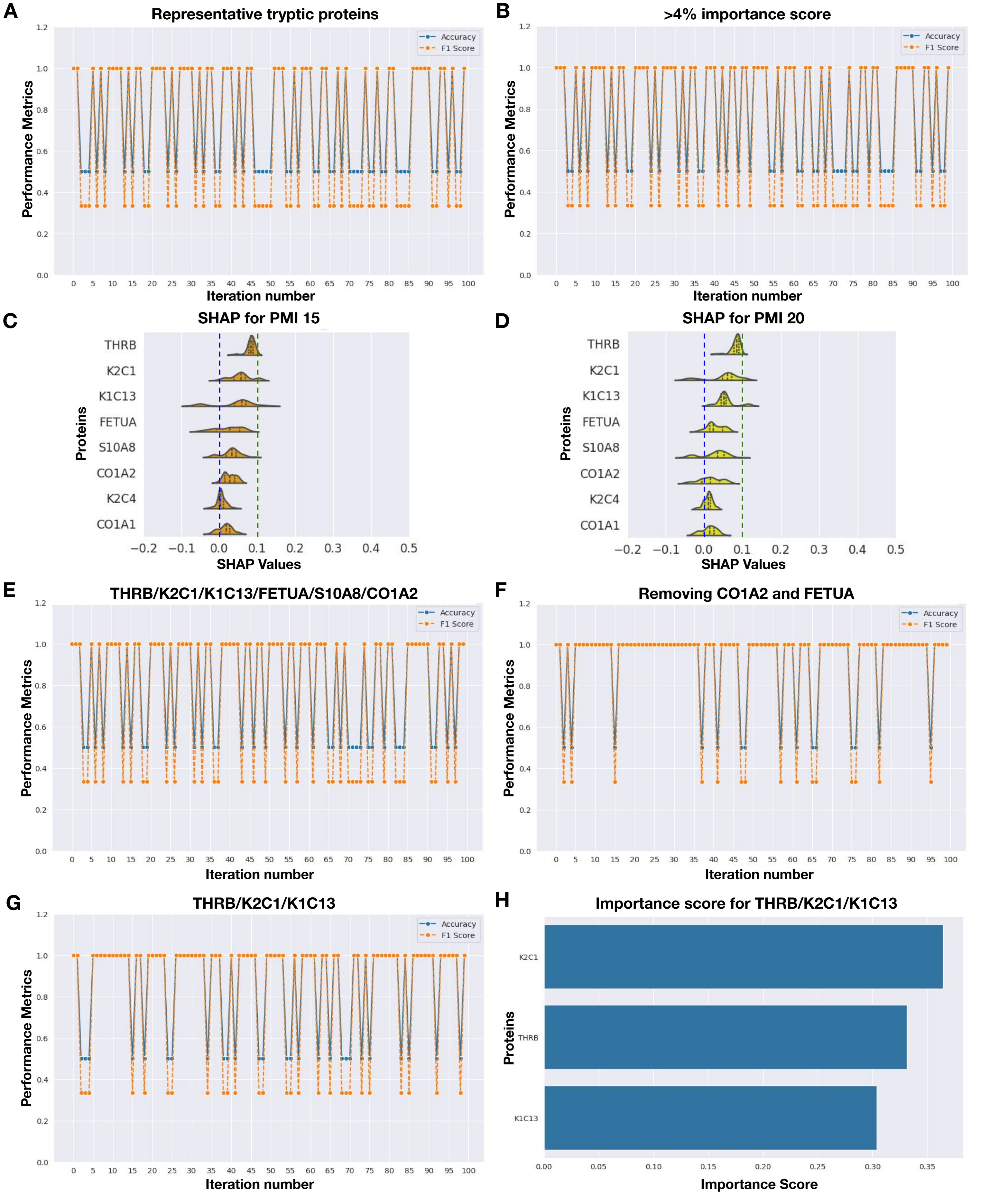

Supplementary Figure 2. Modeling and variable screening for tibia tryptic proteins selected iteratively based on their importance scores and SHAP values. A. Model using identified hyperparameters and the full set of representative proteins. B. Model using eight proteins displaying >4% importance score. C. SHAP values for the 4% most important proteins in the classification of PMI 15 years. D. SHAP values for the 4% most important proteins in the classification of PMI 20 years. E. Model using THRB, K2C1, K1C13, FETUA, S10A8, and CO1A2. F. Proteins CO1A2 and FETUA were discarded from the model E. G. Final model using a minimal set of three proteins: THRB, K2C1, and K1C13. H. Importance score for proteins used in model G.
