# Supplementary material for: Enhancing late postmortem interval prediction: a pilot study integrating proteomics and machine learning to distinguish human bone remains over 15 years
Source: Biol Res. 2024 Oct 24;57:75. doi: 10.1186/s40659-024-00552-8 (PMC11515459; doi:10.1186/s40659-024-00552-8)
Supplement: Supplementary file 3 — Supplementary Figure 3. Variable screening for tibia semitryptic proteins selected iteratively based on their importance scores and SHAP values. A. Model using identified hyperparameters and the full set of representative proteins. B. Model using 11 proteins displaying > 4% importance score. C. Model using PGS1, K1C13, FETUA, K2C1, CO3A1, THRB, and SEMG1. D. Proteins THRB and SEMG1 were discarded for the model in C. E. Subsequent modeling with the elimination of K2C1. F. Final model using the minimal set of three proteins: PGS1, K1C13, and CO3A1. [file 40659_2024_552_MOESM3_ESM.pdf]

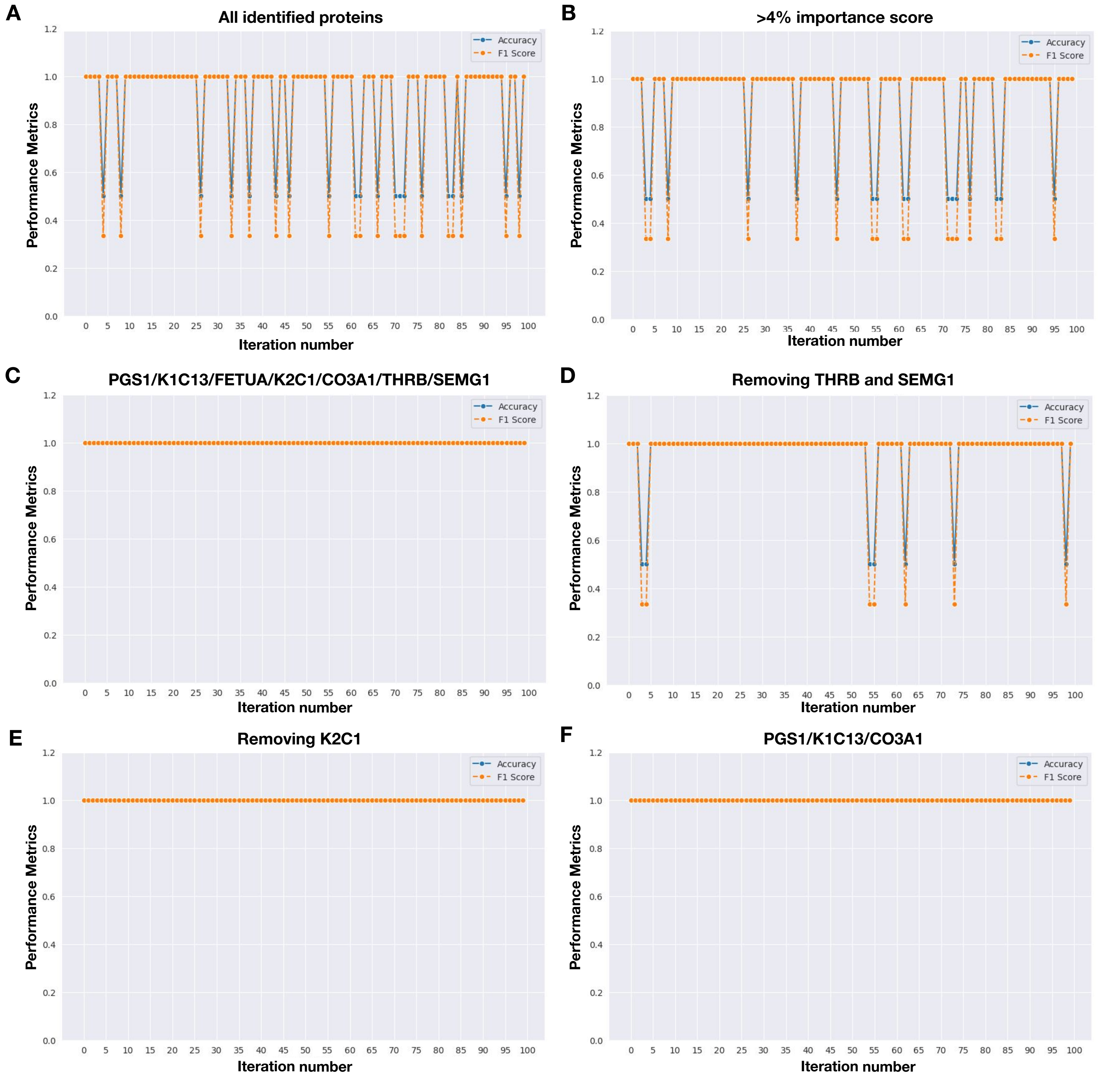

Supplementary Figure 3. Variable screening for tibia semitryptic proteins selected iteratively based on their importance scores and SHAP values.

A. Model using identified hyperparameters and the full set of representative proteins. B. Model using 11 proteins displaying >4% importance score, followed by the elimination of proteins based on SHAP values. C. Model using PGS1, K1C13, FETUA, K2C1, CO3A1, THRB, and SEMG1. D. Proteins THRB and SEMG1 were discarded for the model in C. E. Subsequent modeling with the elimination of K2C1. F. Final model using the minimal set of three proteins: PGS1, K1C13, and CO3A1.
