# Supplementary material for: Enhancing late postmortem interval prediction: a pilot study integrating proteomics and machine learning to distinguish human bone remains over 15 years
Source: Biol Res. 2024 Oct 24;57:75. doi: 10.1186/s40659-024-00552-8 (PMC11515459; doi:10.1186/s40659-024-00552-8)
Supplement: Supplementary file 4 — Supplementary Figure 4. Variable screening for tryptic proteins in ribs selected iteratively based on their importance scores and SHAP values. A. Model using identified hyperparameters and the full set of representative proteins. B. Model using ten proteins displaying > 4% importance score. C. SHAP values for the 4% most important proteins in the classification of PMI 15. D. SHAP values for the 4% most important proteins in the classification of PMI 20. E. Model using K2C1, K2C4, K1C10, S10A9, K1C13, S10A8, and CO1A2. F. Final model using a minimal set of three proteins: K2C1, K2C4, and K1C10. [file 40659_2024_552_MOESM4_ESM.pdf]

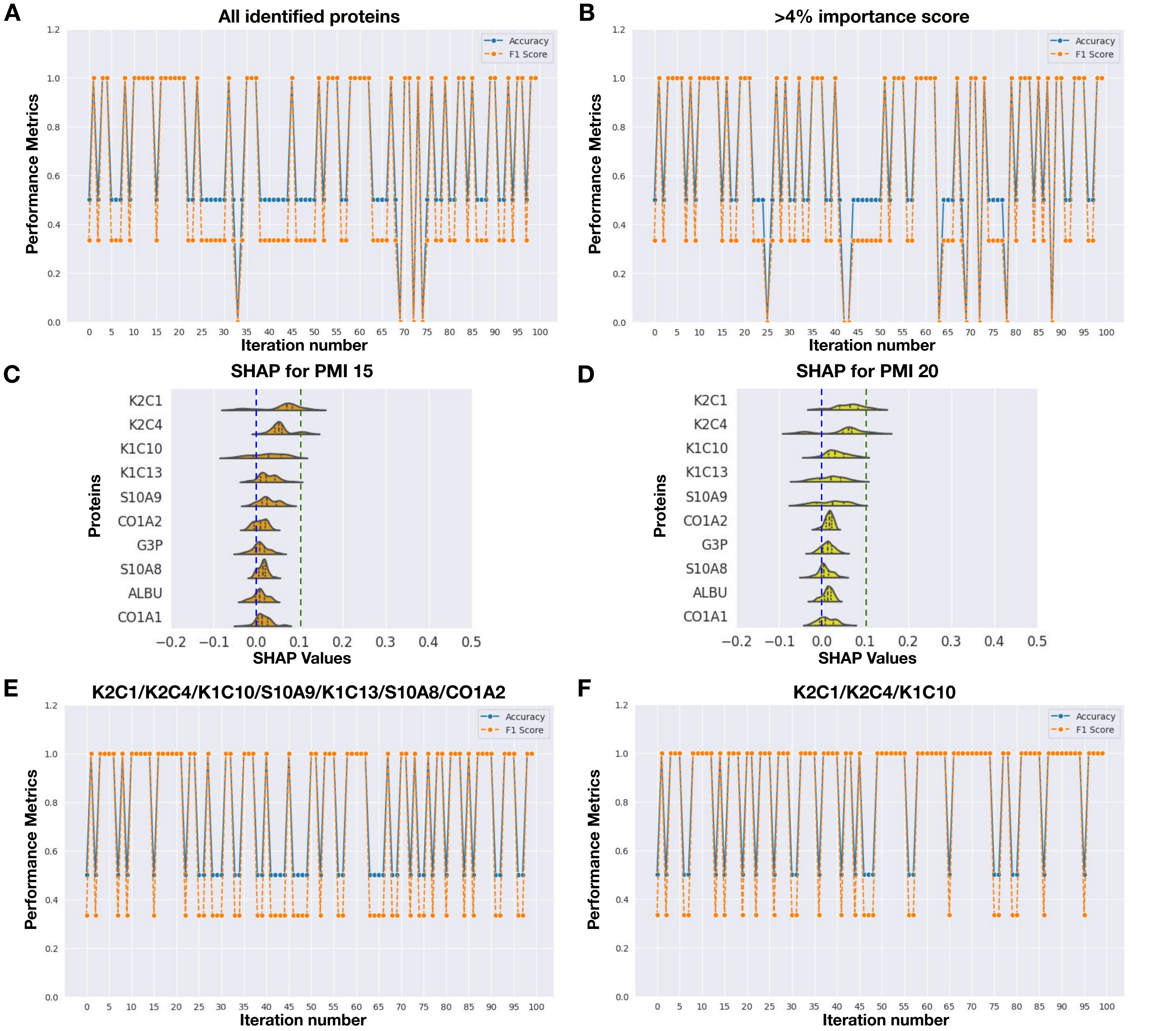

Supplementary Figure 4. Variable screening for tryptic proteins in ribs selected iteratively based on their importance scores and SHAP values.

A. Model using identified hyperparameters and the full set of representative proteins. B. Model using ten proteins displaying >4% importance score. C. SHAP values for the 4% most important proteins in the classification of PMI 15. D. SHAP values for the 4% most important proteins in the classification of PMI 20. E. Model using K2C1, K2C4, K1C10, S10A9, K1C13, S10A8, and CO1A2. F. Final model using a minimal set of three proteins: K2C1, K2C4, and K1C10.
